# Posttraumatic Reactions to Psychosis: A Qualitative Analysis

**DOI:** 10.3389/fpsyt.2017.00129

**Published:** 2017-07-19

**Authors:** Weili Lu, Kim T. Mueser, Stanley D. Rosenberg, Philip T. Yanos, Neisrein Mahmoud

**Affiliations:** ^1^Department of Psychiatric Rehabilitation and Counseling Professions, Rutgers University, Scotch Plains, NJ, United States; ^2^Center for Psychiatric Rehabilitation, Sargent College of Health and Rehabilitation Sciences, Boston University, Boston, MA, United States; ^3^Department of Psychiatry, Dartmouth Medical School, Hanover, NH, United States; ^4^Dartmouth Trauma Intervention Research Center, Lebanon, NH, United States; ^5^Department of Psychology, John Jay College of Criminal Justice, CUNY, New York, NY, United States

**Keywords:** trauma, psychosis, posttraumatic stress disorder, treatment, qualitative analysis

## Abstract

The current study aimed to evaluate the potentially traumatic aspects of psychotic symptoms and psychiatric treatment of psychosis using qualitative methods. Participants included 63 people with first episode psychosis or multiple psychotic episodes recruited from an inpatient psychiatric unit and an urban state psychiatric hospital in the North East region of the United States. Quasi-structured interviews were used to explore those aspects of symptoms and treatment that were perceived as traumatic Emotional reactions to the most traumatic aspect of symptoms and treatment, during and after the event, were also examined. Participants described a number of traumatogenic aspects of psychotic symptoms, including frightening hallucinations; suicidal thought/attempts, thoughts/attempts to hurt others; paranoia/delusions and bizarre/disorganized behavior or catatonia. Traumatic aspects of psychosis elicited emotions including anger, sadness and confusion, anxiety, and numbness at the time of event. Furthermore, many participants found aspects of treatment to be traumatic, including: being forced to stay in the hospital for a long time; experiencing upsetting side-effects; coercive treatments, including involuntary hospitalization, use of restraints, and forced medication; being exposed to aggressive patients; and mistreatment by professionals. These experiences elicited emotions of anger, sadness, distrust, and a sense of helplessness. Study findings suggest that the experiences both of psychotic symptoms and psychiatric treatment, potentially traumatic, can be a powerful barrier to engaging people in mental health services and facilitating recovery. Clinical implications were discussed.

## Introduction

The emergence of a psychotic disorder can be a devastating event for an individual, with major impact on perception of self, self-esteem, and ability to function adequately (1). Psychotic symptoms and treatment experiences can lead to posttraumatic stress disorder (PTSD) symptoms similar to those observed in individuals who have experienced traumatic events such as disasters and rape (2–4). Psychotic symptoms such as command hallucinations to hurt self or others, persecutory delusions, or disorganized behavior can be frightening (2, 4–6). Coercive interventions, such as involuntary hospitalization, seclusion/restraint, and being forced to take medication can be further traumatizing (7–10). These aspects of psychotic episodes are often perceived as threatening and can lead to feelings of fear, helplessness, or horror (4, 11).

The literature regarding psychological reactions to psychosis and its treatment has emphasized both post-psychotic depression and post-psychotic, PTSD symptoms (2, 12, 13). In fact, PTSD is now considered by some as a secondary psychiatric morbidity following psychosis (2). Over 20 years ago, Shaner and Eth (14) reported a case study of a person who developed symptoms, following a schizophrenic episode, that were consistent with PTSD. These symptoms included re-experiencing the traumatic event(s), avoidance of trauma-related stimuli, and over-arousal. Since then, over 17 studies have reported high rates of post-psychotic PTSD symptoms related to these experiences. These 17 studies have included 760 participants, primarily composed of non-affective psychotic disorders (2, 6, 10, 11, 15–27). Using a variety of assessment methods among inpatients and outpatients with psychosis, participants have been asked to respond to the questions based on their reactions toward potentially frightening psychotic symptoms and/or treatment experiences (i.e., negative aspects of treatment). When excluding consideration of whether psychotic or treatment experiences met criterion A for PTSD as defined by DSM-IV, symptoms consistent with a diagnosis of PTSD have been found in 11–67% of the samples, with an average rate of 46%. In addition to traumatic reactions similar to PTSD, depression, suicidality, and low self-esteem are common negative emotional reactions after a psychotic episode.

Post-psychotic depression has been well documented in studies conducted in England (12, 22, 28, 29). It has been found that 36% of patients develop depression following a psychotic episode (12). Depression is common in patients with a first episode psychosis (FEP). Reported prevalence rates varied from 17 to 83% in the different studies (30–33, 34). Relevant studies have generally found that, as treatment progressed, rates of depression decreased. For example, in a sample of 198 Norwegian clients with FEP, 50% of the participants were depressed at the start of treatment, while 35% exhibited depression at one year follow-up after treatment (34). Upon becoming depressed, patients with post-psychotic depression developed lower self-esteem and a worsening of their appraisals of psychosis (35). Additionally, the lifetime risk of suicide in psychotic illness remains high at approximately 7% (29, 36). Suicide risk is highest in the early phases of psychosis (31, 36–39). Upthegrove et al. (36) conducted a study among persons with a first episode of psychosis and found frequent suicidal acts such as overdosing and attempted hanging in this group. Reactions toward psychosis and its treatment are further compounded by the effects of being labeled with a mental illness, rejection and the internalization, acceptance, and resignation to societal stigma toward mental illness (40, 41). Individuals early in the course of psychosis frequently avoid professional help because of concerns about stigma (42), and the negative effects of the internalization of stigma on functioning and well-being have been well-documented [e.g., Ref. (43)].

In the study of psychosis and trauma, there has been significant evidence documenting increased trauma exposure in psychosis and schizophrenia (44–46). Trauma exposure is common among patients with psychosis ranging from 49 to 100% [see, Ref. (46) for a review]: childhood sexual abuse reported by 13 to 64%, childhood physical abuse by 22 to 66%, adulthood sexual assault by 13 to 79%, and adulthood physical assault by 30 to 87% of persons with serious mental illness. Multiple traumatization is common with 75 to 98% of participants report multiple trauma [see, Ref. (46) for a review]. Among 962 participants with psychosis, 17.9% of their sample reported criminal victimization in the past year (47). PTSD is common as a result of trauma exposure among people with psychosis. The current rate of PTSD has been reported as between 25 and 48% in this population in various studies [see, Ref. (46) for a review], nearly ten times higher than that of the general population (48). These traumatic life events, along with the traumatic experience of psychotic episodes and psychiatric treatment, have drawn attention to the need for treatments to reduce the consequences of trauma in this population (49).

Although quantitative literature has documented psychological reactions toward psychosis and its treatment, research is less clear about how psychotic symptoms or coercive treatment experiences are perceived by persons with psychosis. Qualitative research methods are a promising strategy for understanding subjective experience and offering suggestions for potentially useful treatment approaches (42, 50, 51). However, few qualitative inquiries have investigated the subjective experiences of reactions toward psychosis and its treatment.

The current study aimed to evaluate subjective experiences related to psychotic symptoms and treatment in clients with multiple psychotic episodes using qualitative methods. Aspects of symptoms and treatment that were perceived as traumatic, as well as emotional reactions to the most traumatic aspect of symptoms and treatment, were explored.

## Materials and Methods

The current investigation is part of a study evaluating prevalence of PTSD symptoms in FEP or multi-episode participants with psychosis (6, 10). The study was conducted at an inpatient psychiatric unit at a general hospital affiliated with a medical school in North East region as well as an urban state hospital which included both acute inpatient care, as well as intermediate and longer-term treatment. Treatment team members (attending psychiatrists, psychologists, and nurses) were informed about the study and eligibility criteria and then identified potentially eligible participants and referred them to the study team. An important question addressed in the study was the importance of the A1/A2 criteria for traumatic event in diagnosing PTSD related to a psychotic episode. For this reason, we chose to assess participants as soon as possible after the symptoms of their episode had been stabilized to enhance the accuracy of their perceptions and emotional reactions during the index events. Participants were referred to the study after they were judged to be symptomatically stable by their treatment team, and able to provide consent. All of the study procedures were approved of by the appropriate university and hospital Institutional Review Boards.

### Participants

Inclusion criteria for participation in the study were:

(a) age 18 and above;
(b) chart or clinician diagnosis of schizophrenia, schizoaffective disorder, schizophreniform disorder, bipolar disorder with psychotic features, major depression with psychotic features, brief reactive psychosis, or unspecified psychosis;
(c) presentation for treatment of a psychotic episode within the past 6 weeks;
(d) psychotic symptoms of moderate severity or greater on any item on the Brief Psychiatric Rating Scale (52) thought disturbance subscale which includes hallucinatory behavior, unusual thought content, grandiosity, and suspiciousness persisting for at least 2 days in the absence of substance use (53).
(e) history of treatment for at least one psychotic episode including the current episode. A psychotic episode was defined as an episode in which there was the presence of one (or more) of the following symptoms: delusions, hallucinations, disorganized speech, or grossly disorganized or catatonic behavior for at least one week; and
(f) voluntary signed informed consent to participate in the study.

A total of 63 individuals met eligibility criteria and agreed to participate in the study. The characteristics of the study sample are summarized in Table 1. Participants tended to be in their 30s, with an average of over eight past hospitalizations, with 54% of participants were diagnosed with schizophrenia or schizoaffective disorders. A large proportion of the participants in this study were never married (76.9%), and the majority of the participants were African–American or Latino/a.

**Table 1:** Demographic and diagnostic characteristics, and trauma history of study sample (*N* = 63).

	*N*	%
**Gender**		
Male	36	57.1
Female	27	41.9
**Race/ethnicity**		
White	24	38.1
African American	20	31.7
Hispanic	16	25.4
Other	3	4.8
**Marital status**		
Never married	50	79.4
Married	6	9.5
Separated/divorced	7	11.1
**Work status before hospitalization**		
Not employed	46	73.0
Employed	16	25.4
Missing	1	1.6
**Diagnosis**		
Bipolar disorder	17	27.0
Psychotic or delusional disorder	10	15.6
Schizoaffective disorder	12	19.0
Schizophrenia	22	34.9
Other mood disorders	2	3.2
**Traumatic event (yes)**		
Serious accident	28	44.4
Natural disaster	13	20.6
Physical assault by family	37	58.7
Physical assault by stranger	30	47.6
Sexual assault by family	23	36.5
Sexual assault by stranger	21	33.3
Military combat/war zone	1	1.6
Sexual contact younger than 18	27	42.9
Imprisonment	16	25.4
Torture	16	25.4
Life-threatening illness	3	4.8
Other traumatic event	27	42.9
	**Mean**	**SD**
Age	34.43	11.74
Highest grade completed	11.92	2.66
Total # of hospitalization	8.54	7.03
Total # of psychotic episodes	8.49	7.14

### Measures

#### Trauma and PTSD Symptoms

Lifetime exposure to trauma (e.g., sexual assault, serious accident) was evaluated with an abbreviated version of the Traumatic Life Events Questionnaire (54) that included 12 items, each rated on a no/yes basis. After completion of the questionnaire, the participant was oriented to the assessment of psychologically traumatic events related to psychotic symptoms or treatment experiences with the following statement: “I would now like to spend a few minutes finding out about your experiences with psychiatric symptoms.” Interviewers were instructed to adopt the participant’s language when referring to psychiatric symptoms and psychiatric illnesses, such as using terms like “nervous breakdown,” “mental illness,” “stress reaction,” or “emotional upset.” A modified version of the PTSD Assessment Tool for Schizophrenia (PATS), a semi-structured interview designed to elicit posttraumatic reactions to psychosis and treatment experiences (55), was used to guide the discussion. The modified PATS was divided into two sections: reactions to psychotic symptoms; and reactions to treatment experiences. For the first section, reactions to psychotic symptoms were assessed by initially asking a series of 15 questions (e.g., “Have the symptoms of your psychiatric illness ever caused you to feel extremely anxious or terrified?” “Did you believe that groups of people wanted to hurt you?”). An affirmative response to any item was followed up by probes to elicit specific examples. After these questions, participants were asked to identify which experience across all their episodes was most distressing when they looked back on it. Additional questions were then directed at eliciting further details of the event (e.g., when and where it happened, other people who were involved) and how the person reacted to it at the time. Respondents were asked directly: “How did you respond emotionally,” and “What was that like?” Participants were also asked about their immediate and subsequent emotional reactions after the event by inquiring: “What about after the event, how did you respond emotionally?”

Reactions to treatment experiences were assessed in a similar fashion, by initially asking a series of questions (nine in total; e.g., “Have you ever been given a treatment that frightened you?” “Have you ever been forcibly taken to the hospital or to jail?”). Any affirmative response was followed up by questions to elicit specific examples. After these questions, participants were asked to identify the most distressing treatment experience, followed by asking questions aimed at understanding what happened and the person’s reactions to the event both at the time of the event and after the event.

Following the end of the qualitative-portion of the interview, standardized assessments were completed to evaluate PTSD and other symptoms, which have been previously reported (6). The qualitative portion of the interview ranged in length from 15 to 35 min. Field notes by the interviews were used to maintain a record of responses.

### Procedures

Study participants were identified by the clinical treatment team. When potentially eligible patients were identified and were symptomatically stable, permission was obtained from the client for a research team member to discuss the study with them. If permission was granted, a meeting was set up between a research team member and the client, the study was explained, and if the client was interested he or she provided signed informed consent. An interview was then arranged to conduct the assessment, and a chart review was performed. Patients were paid for their participation in the study.

### Analysis

The data analysis for this study was informed by grounded theory, which seeks to generate theory from a data set and is utilized to generate hypotheses (56, 57). The data analysis process in this investigation consisted of an initial open coding of the participant’s responses from the field notes into initial categories by two graduate students. Three raters reviewed the data and the initial categories. They performed axial coding and deleted or combined several of the original codes. After several iterations, a final selective coding process among reviewers which determined the core themes and concepts was implemented (56, 57). Two raters rated the responses independently using the coding theme. The differences and discrepancies were discussed and reconciled after clarifying the original coding scheme.

## Results

Themes specifically regarding one’s perception and emotional reactions toward psychosis and one’s encounters with the mental health system are presented here. Participants described a number of traumatogenic aspects of psychotic symptoms. Five themes concerning traumatogenic aspects of psychosis emerged (see Table 2): (1) frightening hallucinations, reported by 23.8% of the participants; (2) paranoia/delusions, reported by 20.6% of the participants; (3) suicidal thoughts/attempts, reported by 15.9% of the participants; (4) thoughts/attempts to hurt others, reported by 9.5% of the participants; (5) bizarre/disorganized behavior or catatonia; reported by 7.9% of the participants. Other categories included various themes such as flashbacks of past abuse, panic attacks, mania, loss of child custody, etc., were reported by 22.2% of the participants. The most commonly reported theme of traumatogenic aspects of psychosis was that of frightening hallucinations including command hallucinations or persistent hallucinations that last for years, followed by thought/attempts to hurt self, and next by thought/attempts to hurt others. A patient reporting a frightening hallucination (the most commonly identified trauma) is quoted below:

[The most traumatic part is] hearing voices telling me where to move. Three years ago I started to hear voices on TV and radio, telling me to move. I moved non-stop in the past two years, not finding a place to live. People on radio and TV suggest places for me to go.

**Table 2:** Themes and illustrative quotations from personal narratives of psychotic symptoms.

**Frightening hallucinations**
“Voices telling me something bad is going to happen to you.” “Hearing voices that say ‘you are gay’.”
**Thoughts/attempts to hurt self**
“I wanted to die no girl-friend, took a lot of pills that could kill 20 people.” “I heard things, I heard devil telling me to do it I did it before when I was sad, he begged me that I could not do it and survive it and I did it again.” “I tried to hang myself.”
**Thoughts/attempts to hurt others**
“I was obsessed with killing people.” “I wanted to kill everyone who give me harm, like my abusive grandmother.”
**Paranoid/Delusions/grandiose delusions (i.e., feeling like God)**
“I had a suspicion that my aunt who is a psychologist want to hurt me. She knows black magic.” “Going to jail, [there I was feeling that] people trying to poison me.”
**Bizarre behavior/disorganized behavior or catatonia**
“One afternoon, i took my clothes off, I was crazy, ran in the street for 2 miles, people were laughing at me, police took me to hospital.” “I was catatonic, I could not walk or talk, that was traumatic.”

Another participant described how hearing critical voices led to thoughts which led to self-harm:

I was just feeling very guilty about not being a good mother and a good wife. I just felt very bad, and did not think I should be allowed to live, so that’s when I decided that I should hurt myself.

In terms of having experienced thoughts/attempts to hurt self as the most traumatic aspect of symptoms, one participant reported, “I don’t think about killing myself but killing comes to me.” Another reported, “I tried to hang myself.” Another reported, “During [my] first breakdown, when I thought of running into traffic, [that was most traumatic.]” In terms of having experienced thoughts/attempts to hurt others as the most traumatic aspect of symptoms, one participant reported: “I was obsessed with idea of killing people.” One participant reported experiencing homicidal ideas toward her mother and found it traumatic. Participants also found paranoia/delusions the most disturbing. One reported having experienced people spying on him: “[I felt] people spying on me. It happened gradually, everybody saw signs except me.” Another reported, “I feel like I was going to die. Because my other friends will do anything for money there is a possible thing that I may die. People may set things up to get me before my birthday.” A few participants found their bizarre/disorganized behavior most traumatic. One reported great humiliation at having run through the streets naked. He stated, “One afternoon, I took my clothes off, and ran in the street for two miles. People were laughing at me. The police took me to hospital.” Another reported, “I was catatonic, I could not walk or talk, that was traumatic.”

### Emotions Associated With Traumatic Aspects of Psychosis

Emotions associated with traumatic aspects of psychosis involved anger; sadness; mixed feelings such as confusion, sadness, and anger; numbness; sense of shock and anxiety (Table 3). The most commonly experienced emotion in relation to symptoms was anger (reported by 22.0% of participants), followed by shock/anxiety (reported by 18.6% of participants); mixture of the above feelings, or a mixture of sadness and anger (reported by 16.9%); sadness (reported by 15.3%); and numbness (reported by 11.9%). Other categories were reported by 15.3% of the participants. With regard to anger, one participant reported, “I felt angry, clumsy, and confused. I can’t break the spell.” One reported confusion, “I could not understand why I was doing that.” A few participants reported feeling shocked/nervous about their psychotic symptoms. One stated being horrified. Another reported feeling nervous and out of control. One participant specifically reported feeling sad at hearing voices, stating “[After hearing the voices], I was falling apart, crying, always sleeping, never wanted to go out of the house.” Not all participants reported experiencing emotions, but rather reported feeling numb toward what happened during psychosis. One stated, “I felt emotionless” after slashing wrists. Two participants reported they did not know how they felt.

**Table 3:** Themes and illustrative quotations of emotional reaction to symptoms at the time of psychosis.

**Anger**
“Very angry, I wanted to hurt her.” “Upset, should not happen to me.”
**Sadness**
“I cried a lot, depressed.” “[I felt] sad, confused, inconsolable.”
**Mixed feelings such as confusion, sadness and anger**
“I felt sad and angry.” “It feels a little down, angry.” “I felt angry, clumsy, and confused. I can’t break the spell.”
**Numbness**
“I felt emotionless.” “I felt no emotions.” “I don’t know [how I felt].”
**Nervousness/fear/anxiety**
“Nervous, out of control, felt pressured.” “I knew I was in serious trouble police were almost ready to shoot me.” “I was horrified.” “I felt shocked and betrayed.”

Descriptions of emotion looking back after the episode of psychosis involved a wider range of reactions. These involved sadness (14.5%); relief that symptoms went away (14.5% of participants reported); anxiety (12.9%); neutral feelings (11.2%); mixed emotions such as relief mixed with anxiety (9.7%); anger (9.7%); numbness (9.7%); shame (8.1%) and distrust/helplessness (8.1%). Looking back at the most traumatic aspect of psychosis, participants most commonly expressed experiencing sadness about what had happened. It was interesting that equal number of people reported feeling relieved that symptoms were lessened compared to the number of people who expressed sadness about what had happened. Some participants reported feeling neutral about what happened when looking back. Anxiety, mixed emotions, anger, numbness, shame, distrust, and helplessness were also reported by approximately 8–13% of people for each category.

### Traumatogenic Aspects of Psychiatric Treatment

Participants described a number of traumatogenic aspects of psychiatric treatment (Table 4). Amongst the participants who experienced at least one episode of psychosis, five major themes concerning traumatogenic aspects of treatment emerged: (1) long stay in the hospital; (2) medication side effects; (3)coercive treatments, including involuntary hospitalization, use of restraints, and forced medication, and (4) being with other patients; and (5) mistreatment by professionals, not including the use of coercive treatments. The mostly commonly reported theme of traumatogenic aspects of psychiatric treatment was long stay in the hospital, reported by 25 participants (25.8%). One participant in his early 20s was in the hospital for more than 1 year for his first psychotic episode. Another reported, “Staying here for 9 months; I don’t have an apartment, my mom and I don’t get along; [I don’t have a place to go], it feels ridiculous.” Participants also indicated feeling confused about a long stay in the hospital. One person said, “I don’t understand why I am here; they say I should be here for at least 21 days, but I’ve been here for 12 years; the psychologists say I’ll be here permanently.” Another reported, “Since 2001, [I was] only released for 4 months and [I have been] in the hospital for over 6 years.”

**Table 4:** Themes and illustrative quotations for the most traumatic aspect of treatment.

**Long stay in hospital**
“Being in the hospital for 1 year and 3 months is the most traumatic; the cops thought I was living in my car. I was driving from VA to NJ to be with aunt and uncle. Had a lot of stuff in the car. Got arrested for making a call. The cops took me to hospital and I have been here since.” “Since 2001 only released for 4 months and in the hospital for over 6 years.”
**Coercive treatment**
“The fact that my friend and cops should have been more civil to me rather than forcing me to come to the hospital.” (Involuntary hospitalization) “The fact that I was restrained, three guys restrained me [and] gave me a med.” (Restraints) “Several times, I was physically restrained to take meds; I was held down to take injection.” (Forced medication)
**Medications side effects**
“I had prolixin injection, could not sit or stand still after injection.” “Medicines make me fat and destroyed my beauty.”
**Mistreatment by professionals**
“Staff was taking clothes off for me when I took shower.” “Last hospitalization, I felt harassed by pts’ and staff.”
**Being with other psychiatric patients**
“Being exposed to a whole bunch of very sick people for 2 months.” “Seeing a patient pulling her hair and throwing up was the most traumatic.”

The next most commonly reported traumatic event related to treatment was coercive treatments, reported by 14 patients (22.6%), including involuntary hospitalization, use of restraints, and forced medication. In the category of restraints, one reported, “The fact that I was restrained by three guys was the most traumatic.” In the category of involuntary hospitalization, one reported, “The fact that my friend and cops should have been more civil to me rather than forcing me. My friend called cops who hand-cuffed me, and brought me to the ER. Then they shifted me to psych ward.” In the category of forced medication, participants reported that being forced to take medication could be traumatogenic. One reported, “Having them give me Risperdal every two weeks; When I was in the quiet room, they told me if I don’t take it, then they will force me to take it—restrain me to the bed.”

Side effects of psychotropic medication were the third most commonly reported traumatic aspect of treatment (reported by 12 participants; 19.3%). One participant said, “I had Prolixin injections, [and] could not sit or stand still after injection.” Another said she passed out after taking medication. One stated, “Taking bad medications with side effects [was really traumatic]. The psychiatrist gave me a lot of milligrams of medications. After taking them, I could not walk. I felt like I was drunk. It felt like I was being poisoned by the medications.” Others reported medication side effects of weight gain, sleepiness, and fatigue as the most disturbing aspect of treatment.

Mistreatment by professionals, not including the use of coercive treatments, was reported by 14.5% of participants. One stated, “Verbal abuse by staff [was the most traumatic aspect of treatment]. They make fun of our feelings, make me behave the way I don’t want to. If I don’t keep staff happy they will drop my level like a punishment.”

Last, participants also found being exposed to other patients in the hospital, particularly aggressive patients, was traumatic. This was reported by seven participants (11.2%). One reported, “Seeing a patient pulling her hair and throwing up [was the most traumatic aspect of treatment].” Another reported, “I miss my family, I was physically assaulted by two patients, one punched me and the other squeezed my breast.” One reported, “Being exposed to a whole bunch of very sick people for two months was traumatic. At admission, they would scream, cry really loud, there were fights among the patients.”

### Emotions Associated With Traumatic Aspects of Psychiatric Treatment

Participants described a wide range of emotions related to traumatic treatment experiences (Table 5). Participants frequently expressed feeling angry about their psychiatric treatment (reported by 19.0% of participants). One reported, “I got angry. [I felt] violated.” Some participants reported a mixture of feelings such as feeling upset, sad, and angry at the same time: “I got upset,” or “[I was] mad and depressed.” This was reported by 14.3% of participants. Participants commonly dealt with feelings of sadness related to psychiatric treatment (11.1% reported so). One reported, “I felt frustrated, sad, depressed.” Themes of shock, frustration, and helplessness were described by 14.3% of participants. Fear and anxiety were also experienced by 7.9% of participants. One reported, “I was scared.” Another reported, “[I was] terrified.” Feelings of distrust were reported by 6.3% of participants: “I feel paranoid, feel like things are not going to happen in my way.” Lastly, a sense of numbness was reported by 6.3% of participants.

**Table 5:** Themes and illustrative quotations of emotional reaction to treatment at the time of event.

**Anger**
“I felt angry and hurt.” “Get very angry. Personally [I felt] being violated.”
**Sadness**
“Crying, sad, miserable, wished I would die.”
**Mixed feeling**
“I got upset.” “[I was] mad and depressed.”
**Shock/frustration/helplessness**
“[I felt] helpless.” “[I was] shocked.”
**Fear/anxiety**
“Scared, loss of dignity.” “[I was] terrified.”
**Paranoia/distrust**
“I could have reacted worse. I wanted to contact attorney.” “I feel paranoid, feel like things are not going to happen in my way.” “I feel threatened.”
**Numbness**
“I don’t know. Nothing upsets me anymore.”

When asked to how they responded emotionally subsequently to the most traumatic aspect of the treatment, participants described a wider range of emotions related to traumatic treatment experiences. 10% of participants reported feeling neutral about the most traumatic aspect of treatment, when looking back; 8% reported developing a distrust of the system, while another 8% reported numbness or having no emotions after the event. 8% of participants reported feeling relieved that the worst part of treatment was over, while 7% remained sad at what happened. Another 7% of people reported feeling angry or frustrated with the treatment, while 6% of participants reported helplessness or feeling threatened. Mixed emotions or other emotions were reported by four percent of the participants. In total, approximately one-third of the participants continued to experience negative emotions such as sadness, hurt, anger, feeling violated, distrust, dislike, helplessness, or anxiety regarding treatment received.

## Discussion

This study examined traumatic reactions to the experience of psychosis and associated treatment for people with FEP or multiple episodes of psychosis. It supports the conclusion of previous studies that psychosis itself, and related treatment, can both be traumatic for many people. Most of the participants reported traumatic responses related to both their psychotic symptoms and their treatment experiences, consistent with previous reports [i.e., Ref. (11, 18, 20, 21)]. Participants described a number of traumatogenic aspects of psychotic symptoms, corresponding to five themes: (1) frightening hallucinations, (2) suicidal thought/attempts, (3) thoughts/attempts to hurt others, (4) paranoia/delusions, and (5) bizarre/disorganized behavior or catatonia. Traumatic aspects of psychosis elicited emotions including anger, sadness and confusion, anxiety, and numbness. Furthermore, many participants found aspects of treatment to be traumatic, including being forced to stay in the hospital for a long time, experiencing upsetting side-effects, coercive treatments, including involuntary hospitalization, use of restraints, and forced medication, being exposed to aggressive patients, and mistreatment by professionals, and that these experiences elicited emotions such as anger, sadness, distrust, and a sense of helplessness. These findings suggest that the experiences of both symptoms and treatment as traumatic can be a powerful barrier to engaging people in mental health services and facilitating recovery.

Results document the need to address the issues related to posttraumatic reactions to psychosis in people with multiple psychotic episodes. Our findings can inform the development of treatment that is sensitive to these issues. Specifically, findings suggest the need to validate the intense and negative emotional reactions people frequently experience during a psychotic episode and during treatment episodes. Reactions following the acute psychotic episode were more varied, with a small percentage of people reported relief that the worst part was over, while others remained traumatized about what happened. Psychotherapy can be offered to clients who show evidence of posttraumatic reactions and should address these reactions through validation to alleviate traumatic reactions to psychosis. Findings on aspects of symptoms that can cause traumatogenic reactions among clients could also inform the development of treatment protocols so that patients can know what to expect when going to a psychotic episode. Clients commonly reported negative, often traumatizing treatment experiences. The damaging effects of such experiences on client’s trust of mental health professionals were evident in client interviews. Effective treatment of posttraumatic reactions to psychosis can potentially improve clients’ ability to form more collaborative relationships with treatment providers and to play a more active role in the management of their psychiatric disorder (8, 58). Emerging research addressing post-psychotic depression indicates promising results for cognitive behavior therapy (CBT) as a treatment for traumatic reactions toward psychosis (59). Jackson et al. (59) evaluated a form of CBT in reducing trauma, depression, and low self esteem following a first episode of psychosis, in a randomized controlled trial among 66 patients who had recently experienced a first episode of psychosis. CBT has also been found to be effective in reducing hopelessness, when compared to treatment as usual, among a total of 66 participants recovering from FEP (28). In a third study (60), among 22 people recovering from psychosis, those who wrote about the most stressful aspects of their illness showed fewer traumatic symptoms than those who wrote about emotionally neutral topics. Findings suggest that narrative statements disclosing the most stressful aspects of psychosis may lessen the traumatic impact of psychosis (60). Future research on focused intervention for posttraumatic reactions toward psychosis is needed.

Findings from this study underline some of the complicated issues facing the mental health service system. On one hand, participants reported being traumatized by symptoms of mental illness such as distressing auditory hallucinations, bizarre behavior, and persecutory delusions. Simultaneously participants reported being traumatized by interventions (such as involuntary hospitalization, restraints, and forced medication) designed to alleviate these symptoms. However, in many cases, participants experienced these interventions as humiliating or violations of self. Trauma informed care (61) may offer alternatives to restraints and seclusion. Newer models of treatment that place an emphasis on “shared-decision making,” such as the Open Dialog Model (62), have been recommended for people experiencing a first psychotic episode, but our findings suggest that these approaches may need to be offered for persons experiencing multiple psychotic episodes as well. Shared-decision making in medication management, which emphasizes “partnership between two experts: the client and the practitioner,” provides a model for the client to “assess a treatment’s advantages and disadvantages” in medication management [Ref. (63), p. 1636]. Commonground, a web-based application of shared decision making model, encourages the use of resilient self-care strategy in addition to the use of psychotropic medication. It has been found that when CommonGround is implemented, the use of self-management strategies among clients correlated with less concerns about medication side effect, increased perception that medicines were helping, and improved recovery (64). CommonGround has been implemented successfully in community mental health centers (65) while its implementation in psychiatric hospitals has not been reported in the literature. The adoption of shared decision model may reduce traumatic reactions during treatment and foster better trust between providers and clients.

The limitations of this study include sampling issues and the retrospective nature of the data collected. The inpatient participants in the present study were significantly younger (mean age was 33.78) than studies of multi-episode patients with severe mental illness [e.g., Ref. (44, 66–68)]. The participants also had a higher number of hospitalizations (8.55) upon the time of assessments compared to the participants in other studies of persons with severe mental illness. Participants also were more likely to be diagnosed with schizophrenia or schizoaffective disorders (54%), and to be non-white compared to other studies of persons with severe mental illness. These divergences with previous studies suggest that our findings may not be generalizable to a wider range of persons with psychotic disorders. Additionally, experiences of psychosis and contact with psychiatric services were reported retrospectively, recall bias may have affected the accuracy of people’s recollections about their reactions to psychotic experiences and treatment. Finally, during a psychotic episode such as during the height of hallucinations or delusions, the patient may be unable to distinguish reality from non-reality, or even self from other. Therefore, technically, the experience of delusions or hallucinations may not be considered as a traumatic life event, to the same extent as events such as criminal victimization. For some patients with chronic schizophrenia, there is a possibility that these patients never experienced traumatic life events such as abuse or criminal victimization. While we would caution the distinction between a traumatic life event and a traumatic experience of psychosis, treatment however could be similar. Clinical experience has indicated that PTSD-like symptoms induced by delusions can be similarly treated using CBT. For example, a client experienced the delusion of police “shooting” and “killing” his best friend who “stalked” him. Client held onto this fixed delusion and blamed himself for causing the “death” of his best friend. He in turn suffered PTSD-like symptoms including avoidance, hypervigilance, and re-experiencing symptoms. He was successfully treated with CBT by treating his delusion as though the event was real. Once his beliefs in causing the “death” of his best friend were challenged, his PTSD-like symptoms went away. The fixed delusion no longer significantly impacted his life as his guilt for causing his friend’s “death” went away (Lu, unpublished manuscript). Further research is needed in this regard for trauma informed care for persons with psychosis.

In summary, this qualitative study describes the experiences of people during the episodes of psychosis. We found multiple factors contributing to traumatic dimensions of the experience, including actual or perceived coercive aspects of psychiatric treatment. Further research is needed to determine how mental health services can be improved to reduce illness related trauma and facilitate recovery.

## Ethics Statement

This study was carried out in accordance with the recommendations of Rutgers RBHS Institutional Review Board (previously UMDNJ-Newark IRB) and Dartmouth College Institutional Review Board with written informed consent from all subjects. All subjects gave written informed consent in accordance with the Declaration of Helsinki. The protocol was approved by the Rutgers RBHS Institutional Review Board (previously UMDNJ-Newark IRB) and Dartmouth College Institutional Review Board.

## Author Contributions

WL was the site PI for this project. She supervised the operation the project, collected some of the data herself, supervised the data entry and data analysis. She contributed to the conception and the writing of the manuscript. KM obtained the grant. Was the PI of the grant. He supervised WL in the conduction of the research activities in this grant. He provided the research instruments, research ideas, and supervision for the original project. He also contributed to the writing of the manuscript. SR was the co-PI of the project. He provided the research instruments, research ideas, and supervision for the original project. He also contributed to the writing of the manuscript. PY contributed to the writing of the manuscript. NM contributed to the data analysis of the manuscript, composition of the tables, and the editorial assistance of the manuscript.

## Conflict of Interest Statement

The authors declare that the research was conducted in the absence of any commercial or financial relationships that could be construed as a potential conflict of interest.
